# Rapid Prediction of Treatment Futility of Boceprevir with Peginterferon-Ribavirin for Taiwanese Treatment Experienced Hepatitis C Virus Genotype 1-Infected Patients

**DOI:** 10.1371/journal.pone.0137852

**Published:** 2015-09-14

**Authors:** Chi-Chieh Yang, Wei-Lun Tsai, Wei-Wen Su, Chung-Feng Huang, Pin-Nan Cheng, Ching-Chu Lo, Kuo-Chih Tseng, Lein-Ray Mo, Chun-Hsiang Wang, Shih-Jer Hsu, Hsueh-Chou Lai, Chien-Wei Su, Chun-Jen Liu, Cheng-Yuan Peng, Ming-Lung Yu

**Affiliations:** 1 Division of Gastroenterology, Department of Internal Medicine, Show Chwan Memorial Hospital, Changhua, Taiwan; 2 Division of Gastroenterology, Department of Internal Medicine, Kaohsiung Veterans General Hospital, Kaohsiung, Taiwan; 3 School of Medicine, National Yang-Ming University, Taipei, Taiwan; 4 Division of Gastroenterology and Hepatology, Department of Internal Medicine, Changhua Christian Hospital, Changhua, Taiwan; 5 Hepatobiliary Division, Department of Internal Medicine, Kaohsiung Medical University Hospital, Kaohsiung Medical University, Kaohsiung, Taiwan; 6 Graduate Institute of Clinical Medicine and School of Medicine, College of Medicine, Kaohsiung Medical University, Kaohsiung, Taiwan; 7 Division of Gastroenterology and Hepatology, Department of Internal Medicine, National Cheng Kung University Hospital, Tainan, Taiwan; 8 Division of Gastroenterology, Department of Internal Medicine, St. Martin De Porres Hospital, Chia-Yi, Taiwan; 9 Chung-Jen Junior College of Nursing, Health Sciences and Management and School of Medicine, National Yang-Ming University, Taipei, Taiwan; 10 Department of Internal Medicine, Dalin Tzu Chi Hospital, Buddhist Tzu Chi Medical Foundation, Chia-Yi, Taiwan; 11 School of Medicine, Tzuchi University, Hualien, Taiwan; 12 Division of Gastroenterology, Department of Internal Medicine, E-Da Hospital, Kaohsiung, Taiwan; 13 Department of Hepatogastroenterology, Tainan Municipal Hospital, Tainan, Taiwan; 14 Department of Internal Medicine, National Taiwan University Hospital Yun-Lin Branch, Yun Lin, Taiwan; 15 Division of Hepatology and Gastroenterology, Department of Internal Medicine, China Medical University Hospital, Taichung, Taiwan; 16 School of Chinese Medicine, College of Chinese Medicine, China Medical University, Taichung, Taiwan; 17 Division of Gastroenterology, Department of Medicine, Taipei Veterans General Hospital, Taipei, Taiwan; 18 Graduate Institute of Clinical Medicine, College of Medicine, National Taiwan University, Taipei, Taiwan; 19 Hepatitis Research Center and Department of Internal Medicine National Taiwan University Hospital, Taipei, Taiwan; 20 Division of Hepatogastroenterology, Department of Internal Medicine, China Medical University Hospital, Taichung, Taiwan; 21 School of Medicine, China Medical University, Taichung, Taiwan; 22 Institute of Biomedical Sciences, National Sun Yat-Sen University, Kaohsiung, Taiwan; National Taiwan University Hospital, TAIWAN

## Abstract

The efficacy and safety of the boceprevir (BOC)-containing triple therapy in Taiwanese treatment-experienced patients remains elusive. After 4 weeks of peginterferon/ribavirin lead-in therapy, patients with cirrhosis or previous null-response received triple therapy for 44 weeks; whereas others received 32 weeks of triple therapy followed by 12 weeks of peginterferon/ribavirin therapy. Patients with HCV RNA > 100 IU/mL at week 12 or with detectable HCV RNA at week 24 of treatment were viewed as futile. A total of 123 patients received treatment. The rates of sustained virological response (SVR) and relapse were 66.7% and 8.9%, respectively by using intention-to-treat analysis. Multivariate analysis revealed that factors associated with SVR included HCV-1b (odds ratio [OR]/ 95% confidence intervals [CI]: 19.23/1.76–525.15, P = 0.01), BOC adherence (7.69/1.55–48.78, P = 0.01), serum albumin (OR/CI:6.25/1.14–40.07, P = 0.03) levels and HCV RNA levels (OR/CI:0.34/0.12–0.79, P = 0.01). Twenty-six (21.1%) patients experienced severe adverse events (SAEs). Multivariate analysis revealed that APRI > 1.5 was the single factor associated with occurring SAEs (OR/CI: 3.77/ 0.97–14.98, P = 0.05). Merging the cut-off values of HCV RNA > 7 log IU/mL at baseline and HCV RNA > 6 log IU/mL at week 4 provided the earliest and best combing viral kinetics in predicting week 12/24 futility with the PPV of 100% and accuracy of 93.5%. HCV-1 treatment experienced Taiwanese patients treated with boceprevir-containing triple therapy in real world had comparable efficacy and safety profiles with those reported in clinical trials. Early viral kinetics before week 4 of treatment highly predicted futility at week 12 or 24 of treatment.

## Introduction

The treatment of chronic hepatitis C (CHC) has evolved in the era of direct-acting antiviral (DAA) agents. Despite the drastic innovation of DAA in the treatment of CHC, pegylated interferon (peginterferon) and ribavirin combination therapy remains the standard-of-care in most Asian areas including Taiwan. This is due not only to lack of the availability but also to the cost burden in these areas. A favorable sustained virological response (SVR) rate could be achieved in 70–85% of treatment-naïve patients [1,2] and 60–72% [3,4] of treatment-experienced patients in Taiwan due to the relatively higher rate of favorable interleukin-28B genotype in East Asia.[5–7] Nevertheless, a substantial number of patients remains refractory to peginterferon/ribavirin therapy. Introduction of DAA is essential for this population, particularly for patients with advanced liver disease who are in urgent need of viral eradication.

The introduction of the 1^st^ generation of protease inhibitors, boceprevir and telaprevir, has greatly improved the SVR rate; and an increment in SVR rate of approximately 30% and 40% has been demonstrated in treatment naïve and experienced patients, respectively, as compared with patients receiving peginterferon/ribavirin dual therapy.[8] On the other hand, the triple therapy frequently causes adverse events particularly in cirrhotic patients, which preclude its accessibility in clinical practice.[9,10] Notably, reports regarding the safety and efficacy of boceprevir in Asians are scarce. With the rapid evolution of DAA, more potent agents have come to market.[11–13] The issue of early identification of patients who are not candidates for the 1^st^ wave of DAA is of particular interest.

Recently, the CUPIC study has answered the question on the basis of safety consideration in real world cohorts of western countries.[9,10] We herein enrolled HCV-1 experienced Asian patients who previously failed dual therapy. All patients were retreated with boceprevir containing triple therapy in order to elucidate its use in terms of safety in Taiwanese patients. Apart from the CUPIC study, we also focused on early identification of subjects who were not candidates for the treatment by predicting futility on the basis of efficacy evaluation.

## Methods

The study was approved by the ethics committees of the participating hospitals: Show Chwan Memorial Hospital, Kaohsiung Veterans General Hospital, Changhua Christian Hospital, Kaohsiung Medical University Hospital, National Cheng Kung University Hospital, St. Martin De Porres Hospital, Dalin Tzu Chi Hospital, E-Da Hospital. Tainan Municipal Hospital, National Taiwan University Hospital, China Medical University Hospital, Taipei Veterans General Hospital and National Taiwan University Hospital. The study was carried out according to the guidelines of the International Conference on Harmonization for Good Clinical Practice. All patients gave their written informed consent prior to enrollment.

### Patient selection

A Boceprevir Named Patient program (NPP) for compassionate use prior to registration was conducted in 14 participating hospitals in Taiwan in 2013. HCV genotype 1 (HCV-1) patients who previously failed prior peginterferon/ribavirin dual therapy were enrolled.

Previous treatment responses of the eligible patients included relapse (defined as HCV RNA undetectable at the end of treatment but reappearance of HCV RNA during follow up), partial response (defined as HCV RNA reduction > 2 log IU/mL at week 12 (W12) but detectable throughout the treatment period), and null response (defined as < 2 log IU/mL viral reduction at W12 of treatment). Patients were excluded if they had co-infection with human immunodeficiency virus or hepatitis B virus dual infection, history of receiving other direct anti-viral agents, overt liver decompensation, history of organ transplantation other than cornea or hairs, hemoglobin < 12 gm/dL for females and < 13 gm/dL for males, neutrophil < 1500 mm^3^, and platelets < 90,000 mm^3^.

### Regimen

Subcutaneous peginterferon alfa-2b (1.5 μg/kg/week) plus oral ribavirin based on body weight (1000 mg/d for weight < 75 kg and 1200 mg/d for weight ≥ 75 kg) were prescribed in addition to boceprevir (800 mg, 3 times daily). After 4 weeks of peginterferon/ribavirin lead-in therapy, patients with cirrhosis or previous null-response received triple therapy for 44 weeks; whereas others received 32 weeks of triple therapy followed by 12 weeks of peginterferon/ribavirin therapy.

Patients with HCV RNA >100 IU/mL at week 12 (W12) or with detectable HCV RNA at W24 of treatment were viewed as futile.[14] Dose modification of ribavirin was based on the instruction of the manufacture if hemoglobin level was < 10 g/dL, and the use of erythropoietin was at the discretion of the investigators.

### Laboratory testing

The serum HCV RNA at baseline, treatment W4, W8, W12, and W24 were determined by standardized automated real-time PCR assays (Roche Cobas Taqman HCV v2.0 Roche Diagnostics GmbH, Mannheim, Germany) with a detection limit of 15 IU/mL; or RealTime HCV (Abbott Molecular, Des Plaines IL, USA) with a detection limit of 12 IU/ml).[15]

HCV genotype was determined by a reverse hybridization assay (Versant HCV Genotype 2.0 assay, Siemens Healthcare Diagnostics, Illinois) or a real-time PCR assay (Abbott RealTime HCV Genotype II; Abbott Molecular, Des Plaines IL, USA).[16] The sustained virological response (SVR) was defined as seronegativity of HCV RNA throughout 24 weeks of post-treatment follow-up period.

### Statistical analysis

The safety profile assessments, including data on all adverse events, were collected. Efficacy that evaluated virological responses was performed on an intent-to-treat basis. Since HCV RNA levels at W8 of treatment were not routinely checked at each participating site, only patients with available HCV RNA data were evaluated for W8 virological response. Frequency was compared between groups using the χ^2^ test, with the Yates correction, or Fisher exact test. Group means, represented as mean values ± standard deviation, were compared using analysis of variance and the Student *t* test or Mann-Whitney U test. Serum HCV RNA levels were expressed after logarithmic transformation of original values. The aspartate aminotransferase (AST)-to-platelet ratio index (APRI), representing the severity of liver fibrosis, was determined by the following equation: (AST level/upper limit of normal range)/platelet counts (10^9^/L) x 100.[17] The area under the curve (AUC) was compared using receiver operating characteristic (ROC) analysis. An attempt was made to derive a suitable clinical cutoff of HCV RNA levels that would best predict futility. The cutoff point was determined by choosing the point on the ROC curve with the closest distance to the (0,1) point. Multivariate analysis was performed to judge the factors associated with occurrence of severe adverse events (SAEs) and SVR. All statistical analyses were performed using the SPSS 12.0 statistical package (SPSS, Chicago, IL, USA) and were based on two-sided hypothesis tests with a significance level of P < 0.05.

## Results

### Patients

**Fig 1:**
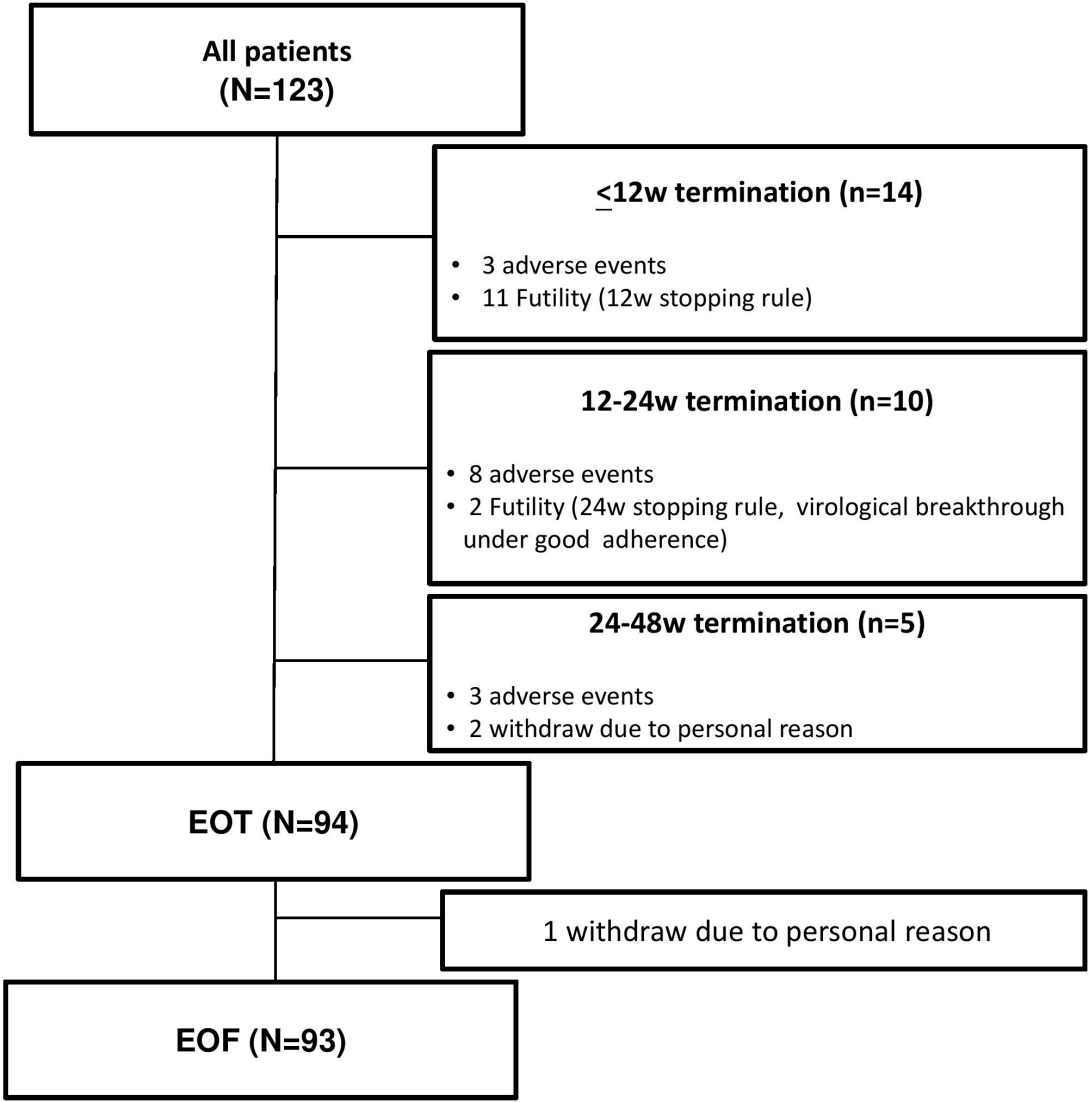
Study protocol. EOT: end of treatment. EOF: end of follow-up, defined as 24 weeks after stopping antiviral therapy.

A total of 123 patients (mean age, 56.7 years; 63.4% males) were recruited. The demographics, virological, and clinical features of the 123 patients are shown in Table 1. The mean HCV RNA levels were 6.3 log IU/ml. The majority of patients (91.9%) were infected with HCV genotype 1b and 34 (27.6%) patients were cirrhotic. Previous treatment responses, including relapse, partial response, and null response, accounted for 55.3%, 18.7%, and 26.0%, respectively, of the study population. Twenty-nine patients terminated treatment before end-of-treatment. Among them, fourteen (11.4%) were due to adverse events, thirteen (10.6%) were due to virological futility, and 2 patients withdrew treatment due to personal reasons (Fig 1).

**Table 1:** Characteristics of 123 chronic hepatitis C patients who failed previous interferon-based therapy. Abbreviations: HCV, hepatitis C virus; AST, aspartate aminotransferase; ALT, alanine aminotransferase.

Male gender, n (%)	78 (63.4)
Age, yrs (mean±SD)	56.7±9.2
Weight, kg (mean±SD)	68.9±11.5
HCV RNA, log IU/mL	6.3±0.8
Hemoglobin, g/dL (mean±SD)	14.8±1.5
White blood cell count, μ/L (mean±SD)	5689±1537
Platelet count, x1000 /mm^3^ (mean±SD)	157±52
Serum albumin, g/dL (mean±SD)	4.2±0.4
AST, IU/L (mean±SD)	80.4±52.9
ALT, IU/L (mean±SD)	103.7±72.5
Creatinine, mg/dL(mean±SD)	0.89±0.20
HCV-1 subtype, n (%)	
1a	5 (4.1)
1b	113 (91.9)
1a+1b	2 (1.6)
uncertain subtype	3 (2.4)
Cirrhosis, n (%)	
yes	34 (27.6)
no	89 (72.4)
Prior treatment response, n (%)	
Relapse	68 (55.3)
Partial response	23 (18.7)
Null response	32 (26.0)

### Safety

The safety profile of the 123 patients is shown in Table 2. Nineteen (15.4%) patients had missed at least one dosage of boceprevir due to non-adherence. SAEs occurred in 26 (21.1%) patients (S1 Table). Univariate analysis of baseline factors associated with SAEs included old age, female gender, higher AST levels, low platelet counts and APRI > 1.5. Logistic regression analysis revealed that APRI > 1.5 was the single factor associated with SAEs (odds ratio [OR]/95% confidence intervals [CI]: 3.77/ 0.97–14.98, P = 0.05) (Table 3). Twenty-four (51.1%) of 47 patients with APRI>1.5 were cirrhotic.

**Table 2:** Safety profile of boceprevir plus peginterferon/ribavirin triple therapy for HCV genotype 1 treatment experienced patients. Abbreviations: AE, serious adverse event.

Events	n (%)
SAE, n (%)	26 (21.1)
Early discontinuation, n (%)	27 (22.0)
Discontinuation due to AE, n (%)	14 (11.4)
Discontinuation due to futility, n (%)	13 (10.6)
Drug modification, n (%)	81 (65.9)
Peginterferon alfa-2b (%)	28 (22.8)
Ribavirin (%)	70 (56.9)
Non-adherence of boceprevir* (%)	19 (15.4)
Laboratory abnormality, n (%)	83 (67.5)
Anemia, n (%)	
8–10 g/dL	62 (50.4)
<8 g/dL	8 (6.5)
Neutropenia, n (%)	
500–750 /mm^3^	16 (13.0)
<500 /mm^3^	2 (1.6)
Thrombocytopenia, n (%)	
20000–50000 /mm^3^	11 (9.5)
<20000 /mm^3^	1 (0.9)
Adverse events, n (%)	
Gastrointestinal symptoms other than dysgeusia	74 (60.2)
Dermatological manifestations	75 (61.0)
Flu-like symptoms	92 (74.8)
Dysgeusia	107 (87.0)
Psychological disorder	60 (48.8)
Hair loss	73 (59.3)

* defined as dose missing during treatment

**Table 3:** Baseline factors associated with occurrence of serious adverse events. Abbreviations: ALT, alanine aminotransferase; Ccr, Creatinine clearance rate; APRI, aspartate aminotransferase-to-platelet ratio index.

	Serious adverse event		Univariate analysis			Multivariate analysis		
	Yes (n = 26)	No (n = 97)	OR	95% C.I.	P value	OR	95% C.I.	P value
Age (mean±SD)	59.3±8.1	56.1±9.4	1.04	1.00–1.12	0.02	1.03	0.97–1.09	0.32
Female, n (%)	14 (53.9)	31 (32.0)	2.48	1.03–6.09	0.04	2.02	0.76–5.42	0.16
AST (mean±SD)	102±64	75±49	1.01	1.00–1.02	0.03	1.00	0.99–1.01	0.97
ALT (mean±SD)	117±78	100±117	1.00	1.00–1.01	0.31			
Platelet count (mm^3^)	139±45	162±53	0.99	0.98–1.00	0.05			
APRI ≥ 1.5, n (%)	17 (68.0)	33 (34.4)	4.06	1.63–10.87	0.002	3.77	0.97–14.98	0.05
Baseline HCV RNA (log IU/ml)	6.1±1.1	6.3±0.7	0.71	0.42–1.21	0.20			
Cr, mg/dL(mean±SD)	0.8±0.2	0.9±0.2	0.29	0.023–2.70	0.30			
Ccr, mL/min (mean+SD)	88.7±21.7	82.3±21.9	1.00	0.99–1.02	0.96			
Cirrhosis, n (%)	11 (42.3)	23 (23.7)	2.36	0.94–5.85	0.06			

### Treatment responses and factors associated with SVR

**Fig 2:**
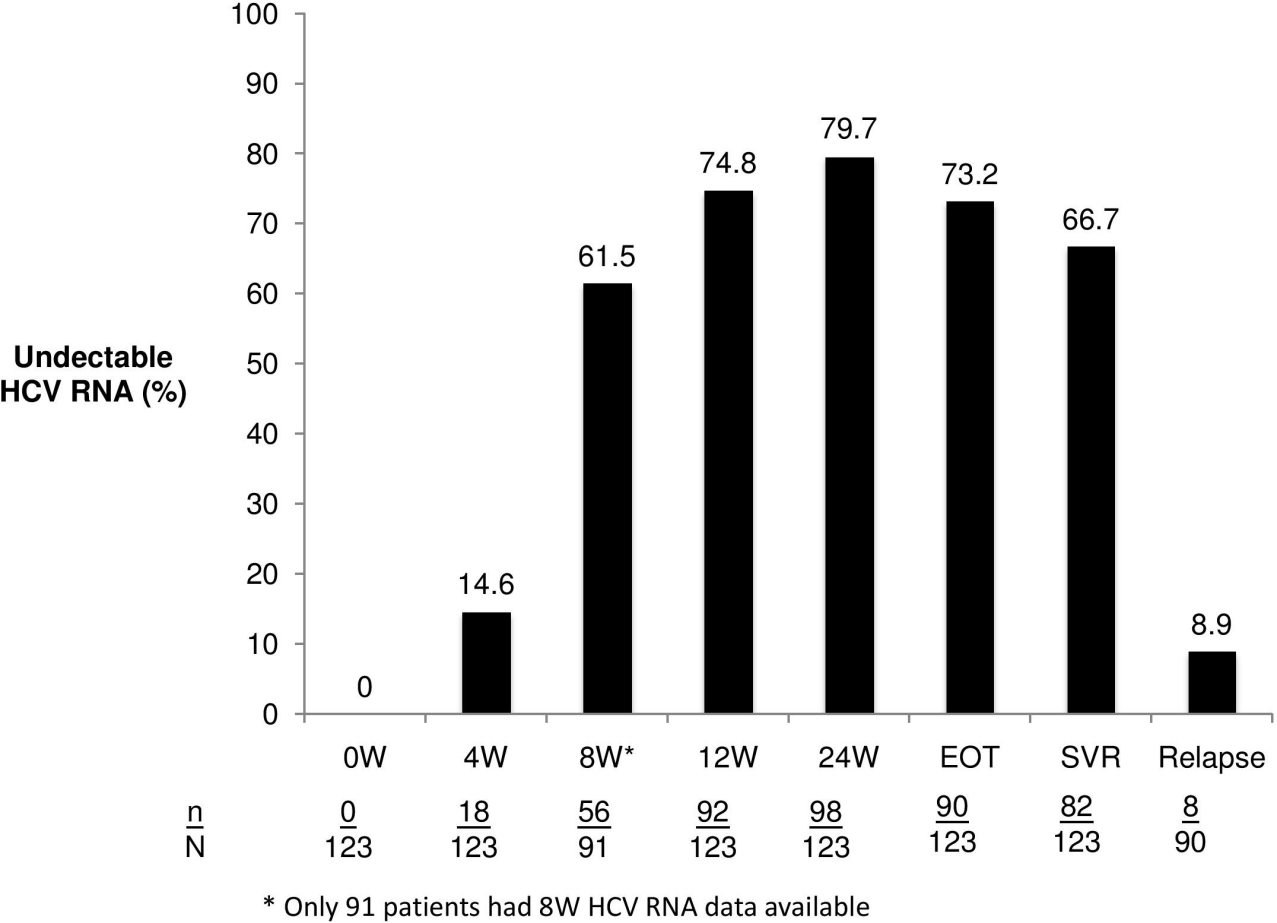
Treatment responses.

By intention-to-treat analysis, the percentages of patients with undetectable HCV RNA at W4, W8, W12 and W24 were 14.6%, 61.5%, 74.8% and 79.7%, respectively. The rates of end of treatment virological response (EOTVR), SVR and relapse were 73.2%, 66.7% and 8.9%, respectively (Fig 2). Univariate analysis of factors with SVR included male gender, low HCV RNA levels, higher platelet counts, higher albumin levels, HCV-1b genotype (compared to HCV-1a), previous relapse (compared to null-response), and BOC adherence. Multivariate analysis revealed that factors associated with SVR included HCV-1b (OR/CI: 19.23/1.76–525.15, P = 0.01), BOC adherence (7.69/1.55–48.78, P = 0.01), serum albumin levels (OR/CI:6.25/1.14–40.07, P = 0.03) and HCV RNA levels (OR/CI:0.34/0.12–0.79, P = 0.01) (Table 4).

**Table 4:** Factors associated with SVR. Abbreviations: SVR, sustained virological response; BOC, Boceprevir; RBV, ribavirin; AST, aspartate aminotransferase; ALT, alanine aminotransferase; Ccr, Creatinine clearance rate; OR, odds ratio; CI, confidence intervals.

	SVR		Univariate analysis			Multivariate analysis (Without BOC adherence)			Multivariate analysis (With BOC adherence)		
	No (n = 41)	Yes (n = 82)	OR	95% CI	P value	OR	95% CI	P value	OR	95% CI	P value
Sex, n (%)											
Female	21 (51.2)	24 (29.3)	1			1			1		
Male	20 (48.8)	58 (70.7)	2.54	1.17–5.56	0.02*	2.27	0.69–7.70	0.18	2.06	0.59–7.41	0.26
Age, yrs (mean±SD)	58.4±9.9	55.9±8.8	0.97	0.93–1.01	0.16						
Weight, kg (mean±SD)	68.4±10.8	69.2±11.8	1.01	0.97–1.04	0.73						
HCV RNA, log IU/mL	6.5±0.6	6.2±0.8	0.51	0.28–0.87	0.02*	0.33	0.13–0.72	0.004*	0.34	0.12–0.79	0.01*
Hemoglobin, g/dL (mean±SD)	14.7±1.5	14.9±1.4	1.10	0.85–1.42	0.47						
White blood cell count, μ/L (mean±SD)	5539±1379	5764±1613	1.00	1.00–1.00	0.45						
Platelet count, x1000 /mm^3^ (mean±SD)	144.4±51.3	163.8±51.7	1.01	1.00–1.02	0.05						
Serum albumin, g/dL (mean±SD)	4.1±0.4	4.3±0.4	6.40	1.85–25.56	0.003*	7.10	1.44–41.85	0.002*	6.25	1.14–40.07	0.03*
AST, IU/L (mean±SD)	91.9±50.0	74.5±53.7	0.99	0.99–1.00	0.09						
ALT, IU/L (mean±SD)	111.9±70.8	99.7±73.5	1.00	0.99–1.00	0.38						
Creatinine, mg/dL(mean±SD)	0.9±0.2	0.9±0.2	1.94	0.27–15.73	0.52						
HCV, n (%)											
1a subtype	4 (10.5)	1 (1.3)	1			1			1		
1b subtype	34 (89.5)	79 (98.8)	9.29	1.32–185.32	0.02*	15.53	1.50–398.04	0.020*	19.23	1.76–525.15	0.01*
Cirrhosis, n (%)											
No	26 (63.4)	63 (76.8)	1								
Yes	15 (36.6)	19 (23.2)	0.52	0.23–1.19	0.12						
Prior treatment response, n (%)											
Relapse	17 (41.5)	51 (62.2)	1						1		
Partial response	8 (19.5)	15 (18.3)	0.63	0.23–1.78	0.37	0.74	0.16–3.65	0.70	0.70	0.14–4.04	0.67
Null response	16 (39.0)	16 (19.5)	0.33	0.14–0.80	0.01*	0.43	0.11–1.56	0.20	0.33	0.08–1.25	0.10
BOC adherence, n (%)											
No	15 (36.6)	4 (4.9)	1						1		
Yes	26 (63.4)	78 (95.1)	4.29	1.16–14.83	0.03*				7.69	1.55–48.78	0.01*

*P<0.05.

### Factors associated with W12/W24 futility

Among the 13 patients who early stopped treatment due to virological failure, eleven had HCV RNA > 100 IU/mL at W12 and the remaining 2 had detectable HCV at W24 (Fig 1). We further evaluated factors associated with W12/W24 futility in the treatment cohort.

### Baseline factors

As shown in Table 5, the association of baseline factors (including demographics, biochemistry, liver disease severity, pretreatment response, and HCV RNA levels) with W12/W24 futility was assessed. Based on univariate analysis, only high HCV RNA level was associated with W12/W24 futility; patients with W12/W24 futility had significantly higher pretreatment HCV RNA levels (6.8 ± 0.5 log IU/mL vs. 6.2 ± 0.8 log IU/mL, P = 0.02). The cut-off HCV RNA level used to distinguish futility was 6.52 IU/mL (AUC 0.73, P = 0.006). Patients with futility had significantly higher proportions of HCV RNA levels > 7 log IU/mL than those without futility (53.9% vs. 17.5%, P = 0.004).

**Table 5:** Factors associated with week 12 and week 24 futilities. Abbreviations: BOC, Boceprevir; RBV, ribavirin; AST, aspartate aminotransferase; ALT, alanine aminotransferase; Ccr, Creatinine clearance rate; APRI, aspartate aminotransferase-to-platelet ratio index; OR, odds ratio; CI, confidence intervals.

	Futility		Univariate analysis		
	Yes (n = 13)	No (n = 110)	OR	95% CI	P value
**Baseline factors**					
Age, yr (mean±SD)	55.1±12.9	56.9±8.7	0.98	0.92–1.04	0.49
Gender, n (%)					
Female	4 (30.8)	41 (37.3)	0.75	0.19–2.46	0.64
Male	9 (69.2)	64 (62.7)	1	-	-
AST, IU/L (mean±SD)	93.8±54.3	78.8±52.8	1.00	0.99–1.01	0.34
ALT, IU/L (mean±SD)	113.9±75.1	102.5±72.5	1.00	0.99–1.01	0.59
APRI ≥1.5	8 (61.5)	42 (38.9)	2.51	0.79–8.81	0.12
<1.5	5 (38.5)	66 (61.1)	1	-	-
Cr, mg/dL (mean±SD)	0.9±0.2	0.9±0.2	1.97	0.09–30.40	0.64
Cirrhosis					
Yes	2 (15.4)	32 (29.1)	0.44	0.07–1.77	0.31
No	11 (84.6)	78 (70.9)	1		
Prior treatment					
null response	6 (46.2)	26 (23.6)	3.69	0.98–15.47	0.15
partial	2 (15.4)	20 (18.2)	2.40	0.44–11.79	0.75
Relapse	5 (38.5)	64 (58.2)	1	-	-
Baseline HCV RNA, log IU/mL (mean±SD)	6.8 (0.5)	6.2 (0.8)	3.63	1.39–11.49	0.02
Baseline HCV RNA, n (%)					
≥ 7 log IU/mL	7 (53.9)	18 (17.5)	5.96	1.79–20.61	0.004
< 7 log IU/mL	6 (46.1)	92 (83.6)	1	-	-
**On-treatment factors**					
BOC dosage (mg/d)	2116±470	2238±462	1.00	1.00–1.00	0.37
RBV dosage (mg/kg/d)	11.6±2.8	12.4±3.6	0.94	0.80–1.11	0.47
4w HCV RNA, n (%)					
≥5 log IU/mL	12 (92.3)	26 (23.6)	38.8	7.13–723.01	<0.0001
<5 log IU/mL	1 (7.7)	84 (76.4)	1	-	-
0–4w RNA reduction, n (%)					
<2 log IU/mL	11 (84.6)	42 (38.2)	8.90	2.25–59.39	0.001
≥2 log IU/mL	2 (15.4)	68 (61.8)	1	-	-
8w HCV RNA, n (%)					
≥2, log IU/mL	8 (100.0)	10 (15.9)	-	-	<0.0001
<2 log IU/mL	0 (0.0)	53 (84.1)	1	-	-
0–8w RNA reduction, n (%)					
<3 log IU/mL	3 (37.5)	2 (3.2)	18.3	2.51–167.57	0.005
≥ 3 log IU/mL	5 (62.5)	61 (96.8)	1	-	-

### On-treatment viral kinetics

As shown in Table 5, both HCV RNA levels and the magnitude of viral reductions at W4 and W8 of treatment were associated with W12/W24 futility. The best cut-off values of absolute HCV RNA levels for predicting W12/W24 futility were > 5.01log IU/mL at W4 (AUC 0.87, P = 0.003) and > 2.49 log IU/mL and W8 (AUC 0.97, P < 0.001), respectively. The best cut-off values of poor viral reduction for predicting W12/W24 futility were < 2.08 log IU/mL decline at W4 (AUC 0.80, P = 0.007) and < 3.47 log IU/mL decline W8 (AUC 0.95, P < 0.001), respectively. Patients with poor W4 and W8 virological response (either high HCV RNA levels or relatively low levels of viral reduction) with different cut-offs had significantly higher rates of futility (Table 5 and Table 6).

**Table 6:** Accuracy of viral kinetics in predicting week 12/24 futility. Abbreviations: AUC, area under the curve; SEN, sensitivity; SPE, specificity; PPV, positive predictive value; NPV, negative predictive value; ACC, accuracy.

	Yes	No		SEN	SPE	NPV	PPV	ACC
	n/N (%)	n/N (%)	P value	(%)	(%)	(%)	(%)	(%)
**Baseline HCV RNA > 7 logs IU/mL**	7/13 (53.9)	18/110 (16.4)	0.002	53.9	83.6	93.9	28.0	80.5
**W4 HCV RNA > 5 logs IU/mL**	12/13 (92.3)	26/110 (23.6)	<0.001	92.3	76.4	98.8	31.6	78.0
**W4 HCV RNA > 6 logs IU/mL**	7/13 (53.9)	4/110 (3.6)	<0.001	53.9	96.4	94.6	63.6	91.9
**W8 HCV RNA > 2 logs IU/mL**	8/8 (100.0)	10/63 (15.9)	<0.001	100.0	84.1	100.0	44.4	85.9
**W4 RNA reduction < 2 logs IU/mL**	11/13 (84.6)	42/110 (38.2)	0.0014	84.6	61.8	97.1	20.8	64.2
**W8 RNA reduction < 3 logs IU/mL**	3/8 (37.5)	2/63 (3.2)	0.0004	37.5	96.8	91.9	60.0	92.4

Since baseline and on-treatment viral kinetics were the major determinants of futility, we final tested the combined effects of viral kinetics in predicting week 12/24 futilities. Merging the cut-off values of HCV RNA > 7 log IU/mL at baseline and HCV RNA > 6 log IU/mL at W4 provided the earliest and best combination of viral kinetics in predicting week 12/24 futility with a PPV of 100% and an accuracy of 93.5% (Table 7). Among 25 patients with baseline HCV RNA level > 7 log IU/mL, 5 (20%) had HCV RNA level > 6 log IU/mL at week 4.

**Table 7:** Combined effects of viral kinetics in predicting week 12/24 futilities. Abbrviations: SEN, sensitivity; SPE, specificity; PPV, positive predictive value; NPV, negative predictive value; ACC, accuracy.

HCV RNA (log IU/mL)	Yes	No	P value	SEN	SPE	NPV	PPV	ACC
	n/N (%)	n/N (%)		(%)	(%)	(%)	(%)	(%)
***At treatment week4***								
**0w RNA > 7 and 4w RNA > 5**	7/13 (53.9)	4/110 (3.6)	<0.0001	53.9	96.4	94.6	63.6	91.9
**0w RNA > 7 and 4w RNA > 6**	5/13 (38.5)	0/110 (0.0)	<0.0001	38.5	100.0	93.2	100.0	93.5
**0w RNA > 7 and 4w RNA decline < 2**	6/13 (46.2)	1/110 (1.0)	<0.0001	46.2	99.1	94.0	85.7	93.5
***At treatment week 8***								
**0w RNA > 7 and 8w RNA > 2**	5/8 (62.5)	0/63 (0.0)	<0.0001	62.5	100.0	95.5	100.0	95.8
**0w RNA > 7 and 8w RNA decline < 3**	2/8 (25.0)	0/59 (0.0)	0.013	25.0	100.0	91.3	100.0	91.5
**0w RNA >7 and (4w RNA >5 or 8w RNA >2)**	6/13 (46.2)	0/103 (0.0)	<0.0001	53.9	96.4	94.6	63.6	91.9

## Discussion

The safety and efficacy of boceprevir treatment in Asian populations has been rarely reported. In the current study, we demonstrated comparable virological responses in Taiwanese patients that were similar to what has been reported in the West.[18] The regimen was also well-tolerated; only one out of ten patients terminated treatment due to adverse events. Instead, about half of the patients who terminated treatment early were due to futility or poor on-treatment responses. Importantly, we identified that early viral kinetics before W8 of treatment highly predicted futility at W12 or W24 of treatment. The prediction could be identified as early as W4 of treatment; all the patients with baseline HCV RNA > 7 log IU/mL and W4 > 6 log IU/mL after peginterferon/ribavirin lead-in encountered W12/W24 futility.

Patient compliance to the regimen in the current study was similar to that found in Western populations.[19] Adherence to treatment determined treatment outcome in patients with peginterferon/ribavirin dual therapy, and it held true for patients receiving the boceprevir-containing regimen.[19] It has been suggested that anemia and dysgeusia were the two most frequent adverse events encountered in the boceprevir-containing regimen as compared with peginterferon/ribavirin dual therapy.[20] Accordingly, we noticed that half of the patients experienced hemoglobin < 10 g/dL, leading to dose reduction of ribavirin. Of note, eight out of ten patients in the current study had dysgeusia, which seemed proportionally higher than what has been reported in the West.[18] Whether a racial difference exists to explain this discrepancy awaits further investigation.

The rate of SAEs was as high as 40.0% in the interim analysis [10] and 49.9% in the final report [9] of the CUPIC cohort. However, the rate of SAEs was only 11.0% (164/1548) in the 3rd phase 2/3 boceprevir registration trials, and drug discontinuation attributed to adverse events was similar in the peginterferon/ribavirin (12%; 67/547) and boceprevir/peginterferon/ribavirin arms (13%; 205/1548).[19] A similar result was observed in the current Asian cohort where about1/10 of the patients discontinued therapy due to adverse events and half of the patients who discontinued therapy did so due to virological futility. The difference in the proportion of patients with advanced liver disease among different studies might have contributed to the difference in the rate of SAEs. Both low albumin level (< 3.5 g/dL) and platelet counts (< 100,000 /mm^3^) were predictive of SAEs among the cirrhotic patients in the CUPIC study [9,10], and a platelet count >100,000 /mm^3^ has been one of the baseline factors associated with an SVR.[9] However, only 1/4 of the patients were cirrhotic and only one patient had a pretreatment level of albumin < 3.5 g/dL in the current study. We did not observe similar results in the CUPIC study. Instead, we found that high APRI was associated with SAEs in the patient cohort with heterogeneous characteristics and these results await replication and further validation.

We have previously shown that poor virological response, in addition to unfavorable interleukin 28B carriage at W4 of treatment, could be viewed as a rapid stopping rule for patients receiving peginterferon/ribavirin dual therapy.[21,22] The adding of boceprevir has broken the rule that a significantly higher proportion of patients could achieve an SVR even if they had had poor virological response after 4 weeks lead-in of peginterferon/ribavirin.[18,23] Instead, the futility rule of boceprevir-containing triple therapy has been refined as HCV RNA > 100 IU/mL at W12 of treatment based on the analysis of SPRINT-2 and RESPOND-2 cohorts.[14] More recently, the stopping rule has moved forward to W8 of therapy. Patients with HCV RNA < 3 log IU/mL reduction had nearly no chance for cure, particularly those with cirrhosis.[24] In addition, we argued that the prediction could advance even more to W4 of treatment. After 4 weeks of peginterferon/ribavirin lead-in therapy, all the patients with baseline HCV RNA > 7 log IU/mL and W4 > 6 log IU/mL would meet the W12/W24 futility rule. For these very difficult-to-treat patients, treatment should be deferred and shift to more potent anti-viral agents.[11] Although many IFN-free DAA regimens are approved for HCV-1 patients in Western countries, most are currently unavailable in Asian countries.[25] Our novel results may guide physicians regarding early treatment decisions or may alter the treatment strategy. In conclusion, HCV-1 treatment experienced Taiwanese patients treated with boceprevir-containing triple therapy in real world had treatment responses and safety profiles comparable with those reported in clinical trials. The early viral kinetics at week 4 highly predicted futility at W12 or W24 of treatment.

## Supporting Information

S1 Table Reasons of occurring severe adverse events. (DOCX)
