# Supplementary material for: Rapid Prediction of Treatment Futility of Boceprevir with Peginterferon-Ribavirin for Taiwanese Treatment Experienced Hepatitis C Virus Genotype 1-Infected Patients
Source: PLoS One. 2015 Sep 14;10(9):e0137852. doi: 10.1371/journal.pone.0137852 (PMC4569190; doi:10.1371/journal.pone.0137852)
Supplement: S1 Table — (DOCX) [file pone.0137852.s001.docx]

supplementary table 1. Reasons of occurring severe adverse events

| **Case No.** | **Age** | **Sex** | **Reasons** |
| --- | --- | --- | --- |
| A8 | 57 | male | skin rash |
| C2 | 50 | female | anemia, traffic accident |
| C3 | 69 | female | anemia |
| C6 | 43 | female | anemia |
| C8 | 63 | male | anemia |
| C10 | 59 | female | severe vomiting |
| C11 | 61 | male | anemia |
| C12 | 55 | female | severe vomiting |
| C13 | 51 | male | anemia |
| C15 | 60 | female | anemia |
| C16 | 51 | male | dizziness |
| C21 | 72 | female | head injury, thrombocytopenia |
| C22 | 66 | male | cellulitis |
| C24 | 69 | female | skin rash |
| C25 | 48 | male | anemia |
| C29 | 62 | female | abdominal distension |
| C30 | 62 | male | depressive disorder |
| D06 | 64 | male | HCC recurrence |
| E03 | 61 | female | hypothyrodism |
| E07 | 66 | male | jaundice |
| E08 | 61 | male | skin rash |
| G01 | 68 | female | urinary tract infection |
| G08 | 64 | female | urosepsis |
| J04 | 53 | female | HCC |
| Y06 | 41 | male | UGI bleeding |
| Y07 | 65 | female | urinary tract infection |
